# Designing a generic, adaptive protocol resource for the measurement of health impact in cash transfer pilot and feasibility studies and trials in high-income countries

**DOI:** 10.1186/s40814-023-01276-4

**Published:** 2023-03-23

**Authors:** Elliott Aidan Johnson, Matthew Thomas Johnson, Christodoulos Kypridemos, Aase Villadsen, Kate E. Pickett

**Affiliations:** 1 Northumbria University, Newcastle Upon Tyne, UK; 2 Social Work, Education and Community Wellbeing, Northumbria University, Newcastle upon Tyne, United Kingdom; 3 Public Health, Policy & Systems, University of Liverpool, Liverpool, UK; 4 Centre for Longitudinal Studies, UCL, London, UK; 5 Epidemiology in the Department of Health Sciences, University of York, York, UK; 6 Centre for Future Health, University of York, York, UK

**Keywords:** Upstream interventions, Cash transfers, Universal basic income, Measures, Pilots

## Abstract

**Introduction:**

In the context of the COVID-19 pandemic, upstream interventions that tackle social determinants of health inequalities have never been more important. Evaluations of upstream cash transfer trials have failed to capture comprehensively the impacts that such systems might have on population health through inadequate design of the interventions themselves and failure to implement consistent, thorough research measures that can be used in microsimulations to model long-term impact. In this article, we describe the process of developing a generic, adaptive protocol resource to address this issue and the challenges involved in that process. The resource is designed for use in high-income countries (HIC) but draws on examples from a UK context to illustrate means of development and deployment. The resource is capable of further adaptation for use in low- and middle-income countries (LMIC). It has particular application for trials of Universal Basic Income but can be adapted to those covering other kinds of cash transfer and welfare system changes.

**Methods:**

We outline two types of prospective intervention based on pilots and trials currently under discussion. In developing the remainder of the resource, we establish six key principles, implement a modular approach based on types of measure and their prospective resource intensity, and source (validated where possible) measures and baseline data primarily from routine collection and large, longitudinal cohort studies. Through these measures, we seek to cover all areas of health impact identified in our theoretical model for use in pilot and feasibility studies.

**Results:**

We find that, in general, self-reported measures alongside routinely collected linked respondent data may provide a feasible means of producing data capable of demonstrating comprehensive health impact. However, we also suggest that, where possible, physiological measures should be included to elucidate underlying biological effects that may not be accurately captured through self-reporting alone and can enable modelling of long-term health outcomes. In addition, accurate self-reported objective income data remains a challenge and requires further development and testing. A process of development and implementation of the resource in pilot and feasibility studies will support assessment of whether or not our proposed health outcome measures are acceptable, feasible and can be used with validity and reliability in the target population.

**Discussion:**

We suggest that while Open Access evaluation instruments are available and usable to measure most constructs of interest, there remain some areas for which further development is necessary. This includes self-reported wellbeing measures that require paid licences but are used in a range of nationally important longitudinal studies instead of Open Access alternatives.

## Key messages

There has been uncertainty about the feasibility of establishing common measures that permit generalisability of findings in specific cash transfer trials—here focusing on Universal Basic Income (UBI) in a UK context—and in development of large, longitudinal datasets, due to the broad range of self-reported and physiological measures currently used. We present measures that enable trials to use existing data as a control and to create data that is generalisable to whole populations *and* can be used to model medium and long-term outcomes.

We have included wellbeing measures that require paid licences but facilitate comparison with existing data. However, we recognise that their use will not be feasible for all studies and therefore offer Open Access alternatives, which may be capable of providing comparable data based on establishing common, evidenced, cut-off points for clinical significance or through their adoption on a widespread basis.

In terms of taking forward findings to the design of pilots and main trials, feasibility studies, including the Welsh Government pilot of basic income for care leavers, will be necessary to establish (a) establish formal power calculations based on the outcomes and demographic groups of interest, and (b) the final costs of the intervention and evaluation, which will determine the specific modules and measures included.

## Introduction

Some 40 years after The Black Report [122] indicated means of affecting social determinants through tax-benefit policy, welfare has failed to promote health. In 2010, 1.3–2.5 million extra years of life and 2.8 million free of illness or disability were being lost annually in England due to health inequalities ([67], 19). Providing support for theoretical work by Grover [32], IPPR [36] attributed 130,000 preventable deaths between 2012 and 2017 to austerity measures. Health inequalities are worsening ([66], 149) and key academic ([110]) and policymaking organisations (EHRC: [37] have lobbied for evidence-based reforms to welfare to promote public health. The COVID-19 pandemic has only increased the urgency of this work.

One of the key under-researched alternatives to the existing system of conditional welfare is Universal Basic Income (UBI), a system of universal cash transfers to (usually adult) citizens or, perhaps pragmatically in a UK context, permanent residents. It ensures a minimum income but, unlike the UK’s Universal Credit [30], is not conditional (i.e. depending on meeting criteria such as being unemployed or disabled to receive benefits). UBI has been presented as a prospective public health measure [51] but has not been piloted or trialled in ways that permit development of health impact evidence [46, 54]. We were funded by the Wellcome Trust to develop a generic, adaptive and feasible protocol resource to evaluate health and wellbeing impact comprehensively for two different types of prospective cash transfer experiments: (a) smaller-scale pilots for 18- to 21-year-olds with lower-than-average socioeconomic status (SES), as in the current Basic Income pilot for care leavers in Wales [118], (b a large-scale full trial involving all people in a small town. While the project was commissioned within Wellcome’s Mental Health Priority area and is informed by Wellcome’s ‘Active Ingredients’ [83], the resource seeks to support measurement and evaluation of impact on health and wellbeing more broadly, both because mental health is correlated with physical health and because measuring physical health impact is critical to assessing potential costs and benefits of schemes. The resource is designed for use in high-income countries (HIC) but draws on examples from a UK context to illustrate means of development and deployment. It is particularly applicable to pilot and feasibility studies and trials of UBI, but can be adapted to those covering other kinds of cash transfer and welfare system changes. We do not seek to prescribe particular dimensions ([108], 366–367) for the cash transfer studies that might use the resource, but greater adaptation will be required the fewer the constituent parts of UBI (universality, unconditionality, etc.) are included in the schemes. For example, particular age groups *may* require a focus on particular health conditions. Low- and middle-income countries may need to focus more on access to infrastructure and services as well as material deprivation. A process of development and implementation in pilot and feasibility studies and trials will support assessment of whether or not our proposed health outcome measures are acceptable, feasible and can be used with validity and reliability in the target population.

### Aims and objectives

In this article, we seek to do the following:

1. Set out key principles for the development of protocols for cash transfer pilot and feasibility studies and trials based on previous theoretical contributions
2. Explore known gaps in evidence on cash transfers resembling UBI to identify the need for a consistent protocol resource
3. Investigate whether health effects *should* and *can* be measured with valid and reliable brief instruments in surveys that must cover multiple topics as is typical in cash transfer experiments
4. Outline the resource and feasibility challenges of some measures, particularly physiological, and how a modular approach to measures banks can address this
5. Examine feasibility issues posed by copyright and paid licensing of measures used in large datasets
6. Make the case for bringing widely used measures with paid licencing conditions into the public domain, or identify and implement comparable Open Access alternatives

#### Existing evidence: income, health and welfare

There is a broad body of evidence to indicate a causal relationship between income and health. Systematic reviews have presented evidence of associations between income and inequality as determinants of population health (e.g. [58, 59, 69, 89, 90], child health, wellbeing and educational outcomes [14], and adult mental health [113]. Indeed, supporting Pickett and Wilkinson’s findings [82], Adeline and Delattre [2] endorsed both the Absolute Income Hypothesis (a positive and concave effect of income on health) and the Income Inequality Hypothesis (that income inequalities affect all members of a society). Our previous work [80] analysed data from 10 waves of the Understanding Society UK Household Longitudinal Study and found that each step down in average household income quintile was associated with a higher probability of reporting clinically significant symptoms of anxiety and depression among 16- to 24-year-olds. It also found that increases in income over time were associated with a reduction in that probability. As such, the overwhelming body of evidence supports the notion of an increase in income being the ‘ultimate “multipurpose” policy instrument’ ([68], 145).

**Fig. 1:**
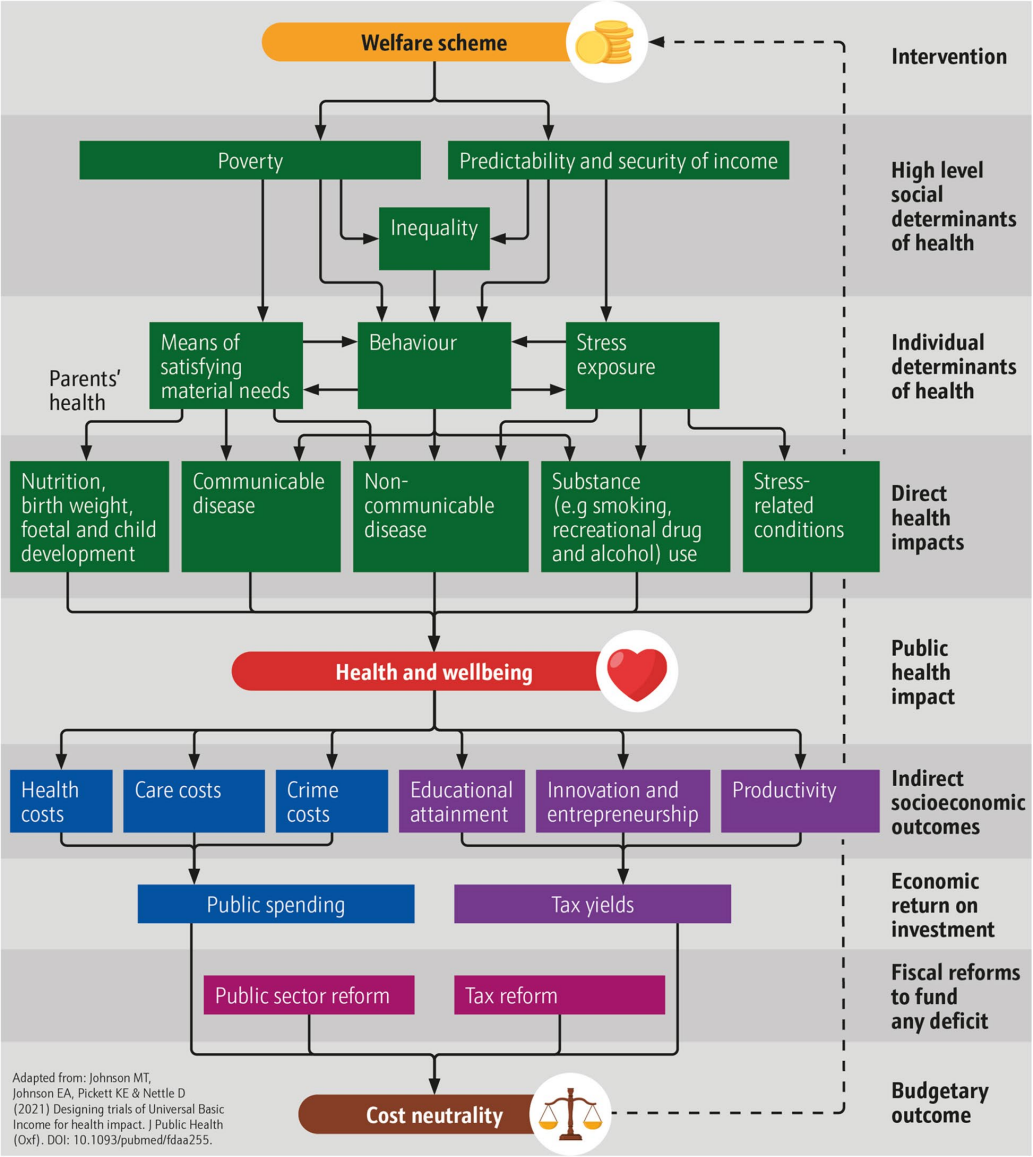
Welfare model of impact (adapted from Johnson M et al. [52])

Crucially, despite the clear evidence of a relationship between income, welfare and income, ([5], 52) have argued that there is ‘less clarity regarding the particular role of income as a health determinant or the mechanisms by which income modification interventions might affect health’. Based on the literature, we have presented three pathways to health through welfare [52], which we represent in Fig. 1. Where welfare increases:

i) Size of income, it can reduce poverty, thereby improving quality of resources by which to satisfy basic needs [50].
ii) Security of income, it can reduce stress associated with exposure to threat of destitution [51].
iii) Predictability of income, it can reduce ‘extrinsic mortality cues’ and promote longer-term thinking conducive to health promoting behaviour (e.g. substance use and relationship formation) [81].

A safety net that reduces ‘health inequalities and the structural conditions that put people “at risk of risks”’ ([112], S47), can, therefore, potentially serve as a significant public health instrument. However, conditional welfare systems like Universal Credit are often associated with poor outcomes. Receipt in high-income countries is associated with worse health outcomes [100], increased psychological distress prevalence [119] and reduction in activity [1, 48]. Our model suggests several explanations: current welfare schemes are ‘insufficient to offset the negative health consequences of severe socioeconomic disadvantage’ [100], conditionality (requirements such as being unemployed or disabled to receive benefits) and assessment inflicts stress [18] and creates perverse incentives for health-diminishing behaviour ([52], 412), and focusing on the poorest fails to mitigate broader determinants that affect society as a whole (see [67], 16). It is for these reasons that organisations, parties and commentators have called for evaluation of alternatives (The [110]).

Evidence on alternative systems, such as UBI, is less clear by virtue of the absence of representative trials and the failure to evaluate health impact in a consistent and generalisable manner within previous cash transfer programmes. Gibson, Hearty and Craig’s [25] scoping review examined interventions similar to basic income. Where transfers reduced poverty, research found increased birth weight [9], illness and injury reduction [4], and decreased hospital admissions [23]. Where schemes reduced conditionality, qualitative studies found improved adult mental health ([34, 55], 24), fibromyalgia and coeliac disease [33]. Where schemes increased predictability of income, studies showed reduced substance misuse [15].

However, the schemes from which the evidence was drawn were unrepresentative of prospective trials in the UK as payments were either not applied to entire populations, were contingent on ethnicity, made to heads of households, were periodic or too small [46, 54]. Moreover, the trial protocols have failed to secure comprehensive generalisable data on health impact for a number of reasons, such as that they (a) focus solely on mental health measures with regard to the role that stress plays rather than accompanying physical health effects; (b) consider outcomes that would be anticipated solely from poverty reduction among the very poorest rather than health impacts across the population; (c) fail to measure a broad range of measures of stress and subjective socioeconomic status that affect those broader sections of society [46, 54]. The consequence of inadequate design and evaluation in previous trials is that assessments of UBI are likely to have underestimated health impacts and overestimated net costs.

#### Microsimulation’s role and requirements

In terms of understanding long-term population-level outcomes, even comprehensive, systematic reviews and meta-analyses of available data, like Romero et al.’s [93], are unable to provide the data required for microsimulation. This is because microsimulation estimates distributional outputs and studies must report not just the average effect of the intervention but the disaggregated distributional effect as well. For example, ideally, data should report the intervention’s differential impact across different age groups, sex, and income deciles. This would enable a more realistic simulation of the potential policy impacts and in-silico experimentation of multiple policy implementations.

With regard to prospective cash transfer systems, randomised controlled trials (RCTs), and other experimental designs, are crucial as they play the following roles:

1. Identify the causal mechanisms between income (including quantity and quality) and health
2. Prove risk reversibility, i.e. that cash transfer interventions can reduce the excess risk of living in material deprivation. This is crucial for policymaking as it would justify cash transfer policies
3. Quantify the impact of the intervention
4. Identify the most effective intervention designs

Given rising interest among policymakers in UBI, as well as other cash-transfer upstream interventions, there is genuine need for research protocols capable of being deployed effectively in different trial conditions. However, it is perhaps unfeasible that a single RCT could fulfil all the roles above because it would require too large a sample size and a long observation period that may not be feasible in the current political and academic environment. Therefore, multiple RCTs may be required to explore the issues and produce much-needed data on efficacy. This makes the consistency of outcome measures between trials essential. In this regard, the abundance of observational and experimental studies requires evidence synthesis. Simulation modelling is uniquely positioned to synthesise all available evidence and estimate what cannot be directly observed. Microsimulation specifically can simulate the causal pathways between income and health and quantify the distributional impact of policy-relevant what-if scenarios.

Quantifying the potential effectiveness, cost-effectiveness, and equity of a proposed cash transfer interventions (e.g. UBI) through modelling requires the simulation of two counterfactual scenarios: the baseline scenario (i.e. UBI is not instituted) and the policy scenario (i.e. UBI is instituted across a nation). The baseline scenario needs to be informed by existing population-representative observational studies, such as longitudinal cohort studies (e.g. Understanding Society and the Millennium Cohort Study). The policy scenario needs to be informed by RCTs of the proposed intervention, although modelling based on observations based on income can aid understanding of the potential impacts cash transfers could have ahead of representative RCTs. Therefore, outcome measures of the RCTs need to be harmonised with the measurement instruments of the population-representative observational studies.

Given this background, there is a need to produce a generic, adaptive protocol resource capable of being deployed in very different types of trial. In this article, we outline development of the resource for two types of trial being considered by policymakers that operate at significantly different scales: (a) smaller-scale pilots for 18- to 20-year-olds in urban areas with lower-than-average socioeconomic status (SES); (b) a large-scale full trial involving all people in a small town. There are tangible proposals relating to these types of trials (see ‘Trial duration and regularity of data collection’ section below), but we wanted the resource to be sufficiently generic and adaptable to be of use in most possible situations, at least within a UK context. The two trial types necessarily differ according to scale and measures used. The former establishes feasibility in terms of ethics, payment and proof of research concept. The latter is necessarily broader as the impact of the intervention would be broader and would focus more clearly on establishing collective-level efficacy and broader socioeconomic outcomes.

## Methods

We have previously established a number of limitations in the existing data [46, 54], guidelines for developing trials designed to promote public health [53] and means of modelling long-term population-level health and economic impacts from trials [53]. Our findings informed some broad underpinning features for pilot design, which we discuss in the results section below. Our development of generic adaptive protocols proceeded according to principles established in [46, 54]:

1. Routine collection ought to be the foundation for baseline comparison of society-level outcomes
2. Measurement ought to capture wellbeing in its broadest form
3. Only measures validated against morbidity and mortality be deployed
4. Self-reporting requires simplicity and limits on respondent load to ensure accuracy
5. Cost ought to be minimised where similar outcomes can be produced via cheaper procedures

We have added a sixth principle to this in light of the risk of research misuse on the subject of welfare (see impact of reforms in [116]:

6. Where possible, questions ought to be the result of co-production with, or reflect the assessment of, people most vulnerable to welfare reforms

Upholding these principles ensures a range of measures are available that provide comparability between data sources and a structure that facilitates use in microsimulation modelling. The design of protocols for adaptive use in cash transfer trials is necessarily generic and broad as projects will vary substantially depending on context aims and resource available. As such, we structured our enquiry around evaluating and collating four modular components for studies:

1. Essential administrative data
2. Available comparative data from routine collection and national surveys
3. Self-reported substantive measures
4. Physiological measures requiring more intensive collection methods and analysis

Given the need for data to administer the intervention and evaluation, control data where interventions have been provided to everyone within a locality, and the need for substantive, efficient, health outcome data, we held a working assumption that modules 1, 2, and 3 would be essential to completion of any study, while module 4 could be included or omitted depending on resourcing.

We began by reviewing the scale, scope and accessibility of data from routine data collection and national surveys such as the Census, Public Health Profiles (and its sources), Family Resources Survey, Crime Survey for England and Wales and NHS activity. We looked at the possibility of using linked patient data, given that it has the potential to reduce respondent load and enable substantial data gathering that might otherwise be required through self-reporting in a trial measuring health outcomes.

We then reviewed the literature to establish the scope, validation and licensing status of survey questions deployed in large longitudinal cohort studies, including the Millennium Cohort Study, Next Steps and Understanding Society. While recognising that results from observational studies may underestimate strength of association ([14], 981), we supplemented the review with statistical analysis of the relationship between some commonly used mental wellbeing measures and diagnosis of anxiety and depression. Using data from Understanding Society (wave 10), our analyses showed that among 14- to 24-year-olds, self-reported diagnosis of anxiety or depression was predicted better by SF-12 (OR 3.12, 95% CI 2.57–3.78) than GHQ-12 (OR 2.18, 95% CI 1.85–2.56), using standardised measures of these predictors to make results comparable. This work served two functions. First, it built into the protocols capacity for comparison with large datasets and, second, it enabled assessment of the viability of adopting solely Open Access questions in order to comply with Principle 5. The cohort studies also provided key demographic and administrative questions that could be employed.

We examined measures requiring an interviewer and sought to identify alternatives suitable for self-reporting. Finally, we looked at physiological measures in order to establish whether there was strong case for their inclusion or whether self-reported alternatives would be sufficient.

In the results below, we have highlighted relevant levels of potential impact from Fig. 1 in parentheses, though some cover more than one input, output or outcome level.

## Results

### Routine collection and baseline data

#### Demographic and economic

We identified several core sources of data from routine collection and national surveys that could be deployed in trials within England, Wales and Scotland. First, UK Census data is available from the Office for National Statistics’ [78] Nomis covering demographic categories (determinant of health) and socioeconomic status (determinant of health and indirect socioeconomic outcome) along with self-rated global health (direct health impact) and social model of disability (determinant of health and public health impact) by small administrative areas. It is, fundamentally, the sole major source of such data that is drawn not from a sample but instead contains responses from almost the whole population. 2011 data is currently available, which, unfortunately, does not cover the substantial changes in socioeconomic circumstances that have taken place under austerity policies of the last decade. Full 2021 Census data will, however, be available from March 2023 [76]. Further official labour market statistics (determinant of health and indirect socioeconomic outcome) are available through Nomis from a range of, usually annual survey, sources, including the Annual Population Survey. Finally, more detailed, and up-to-date socioeconomic data (determinant of health and indirect socioeconomic outcome) is available from the Department for Work and Pensions, Office for National Statistics and NatCen Social Research’s [17] Family Resources Survey, which is of significant importance with regard to tax-benefit microsimulation modelling [91].

#### Routine, population-level health indicators

We also located a number of sources of routine and national survey health data (public health impact). This included the Office for Health Improvement and Disparities’ [75] Public Health Profiles (which collate population health and health behaviour data for England at local authority level), the Scottish Public Health Observatory’s [99] ScotPHO Online Profiles Tool (which presents similar data for Scotland at national, NHS health board or local area level depending on measure) and Public Health Wales’ [86] Observatory which provides similar data but is currently under development. Further physical activity data at local authority level is available for England through Sport England’s [104] Active Lives Online, while the relevant data for Scotland through the Scottish Government’s [98] Scottish Health Survey is only available at national level.

#### Area-level crime

In terms of crime statistics (indirect socioeconomic outcome), recorded crime in England is available by Community Safety Partnership level Office for National Statistics [79]—which broadly equate to local authority areas—and by local authority level in Scotland [96]. Crime and crime perceptions data are available from the Crime Survey for England and Wales at police force area level [77] and the Scottish Crime and Justice Survey at Police Division level [97].

#### Health data for comparison with trials (direct and public health impacts)

The key sources of health data that have the potential to be compared against that produced through cash transfer evaluations form two groups. First, there are large cohort studies, both longitudinal—such as Understanding Society [45], the Millennium Cohort Study [12], Next Steps [13], Whitehall II [19, 40]—and cross-sectional, primarily the Health Survey for England, Scottish Health Survey and Welsh Health Survey. In general, these studies provide large-scale, comprehensive health data from self-reported measures and, in the case of Understanding Society, Whitehall II and ELSA, physiological measures such as biomarkers. Unfortunately, the data from the majority of these studies is often underpowered to explore associations at subnational (or subgroup population) level, with regional data available in the Health Survey for England. It does provide comparison data by demographic groups, however, such as socioeconomic status, so is of significant use in, for example, microsimulation modelling. Most self-reported data is publicly available in some form, while physiological measures sometimes have data-sharing requirements. The second source of health data is NHS activity data at both primary and secondary levels. Tracking changes in activity is possible in England at Clinical Commissioning Group (CCG) level for primary care (NHS [73]) and, to some extent, at secondary care level (NHS [74]). Scottish hospital data are available at NHS Board level [84]. Fewer sources of Open Access primary care data appear available [85]. However, the Clinical Practice Research Datalink [10], which requires a paid licence, provides data based on patient electronic health records from a network of GP practices from across the UK.

#### Linked data at local or regional level

Finally, in some areas, and for some research studies, linking respondent data with their patient records (direct health impacts) or other data sources (e.g. determinants of health or indirect socioeconomic outcomes) may be possible. In Bradford, for example, Connected Bradford [102] has been implemented to streamline this process. In London, the boroughs of Tower Hamlets [111] and Barking and Dagenham ([7], have also created anonymised/pseudonymised datasets drawn from a range of health and local authority data.

With these routine and comparative data sources identified, we moved on to develop the self-reported questions component of the measures bank.

#### Self-reported questions

In considering which measures to include in our bank, we again prioritised those in large, national longitudinal cohort studies, both due to the validation status inherent in such measures and their ability to provide comparative data that can be used in microsimulation modelling to fill any gaps in the evidence collected during trials. In addition to administrative questions, we looked for measures in three broad themes.

#### Demographics (determinants of health)

Due to harmonisation efforts by the Government Statistical Service [31], demographic measures are, on the whole, sufficiently consistent at national statistics level and at least comparable in other large datasets. We therefore prioritised England and Wales 2021 Census measures for this section, particularly as it will provide up-to-date, accurate data at very small administrative area level. While it does not contain comprehensive health and wellbeing data, it is very useful as a means of populating microsimulation models with data that has not been estimated. Some measures, such as gender and assigned sex have been taken from Understanding Society as the previously agreed Census measure guidance was changed by court order [114] resulting in potentially inconsistent wording with regard to sex and gender identity.

From a theoretical perspective, it was important to ensure that measures were included for all potential demographic sources of socially determined inequalities in health. It is plausible that different groups, based on gender identity, cultural background, religion or sexual orientation might be impacted differently by socioeconomic interventions. For example, women and LGBT people might disproportionately benefit from independent economic security that could enable escape from domestic violence or intimidation and secure reduced stress and increased wellbeing and flourishing.

#### Socioeconomic status and household composition (determinants of health and indirect socioeconomic outcomes)

Our review highlighted the difficulty of deploying a single set of questions to establish household and socioeconomic baselines for the broad range of cash transfer trials that might be undertaken. Household grids are used in large surveys like the Census, Family Resources Survey, Millennium Cohort Study and Understanding Society. However, in respect of Principle 4, they are extremely cumbersome and time consuming. For example, Understanding Society’s Household Grid module contains a potential 115 questions [43]. In keeping with Principle 6, co-production with young people as part of the Born in Bradford: Age of Wonder project, resulted in the development of a three-question household composition question. In that project, however, evaluation is primarily focused on individual young participants. A cash transfer trial may look at impacts on one individual in a household alone (as in [56], 52), but, as we have argued [46, 54], it is important to consider the effect of such interventions on households, communities and society as a whole. Measurement on the basis of heads of household alone is likely to replicate issues identified in several previous interventions. For the measures bank, we developed a new grid system for use online for completion by a head of household that facilitates cascading individual questionnaires. In future testing, we intend to undertake primary research and co-production to understand the impact on respondent load and response accuracy of these options.

A factor in the need to reduce respondent load and simplify administration is measurement of objective and subjective socioeconomic status (SES). Although an individual is unlikely to answer all of these, Understanding Society [44] has a total of 169 possible questions relating to SES. That study is sufficiently large and well-funded to support this kind of administration. However, for smaller projects, and even the larger of our two theoretical studies in which more than annual collection would be needed, this is unlikely to be feasible. We therefore decided to focus on the most fundamental and replicable measures of SES based on our analysis of datasets and the requirements we have identified for modelling [62].

We have shown that within- and between-individual variations in net equivalised household income are associated with greater prevalence of clinical-threshold level symptoms of poor mental health through measures such as SF-12 [80]. We therefore developed a simplified measure of household income based on the Institute for Fiscal Studies’ [41] ‘Your household's income: Where do you fit in?’ tool. This requires 10 questions to be answered by the head of household. We also include guidance about calculating net income for self-employed people and questions on receipt of benefits, since engagement with welfare has a substantial relationship with the subject of cash transfers. These aim to provide simplified versions of the Before Housing Costs and After Housing Costs measures, with the latter requiring some imputation from national data. Due to the complexity of the requirements above, they are not completely comparable with national data. The DWP’s After Housing Costs measure, in particular, is likely to result in significant respondent load as it requires calculation of, for example, mortgage interest but not balance repayment. Further work and testing is required to identify whether inclusion of measures that wholly reflect national data is possible.

Importantly, we include subjective SES questions from the Millennium Cohort Study (MCS) associated with poorer mental wellbeing. In young people aged 16–24, the MCS measures were more monotonically associated with poor mental health than average household income [115]. They were also strongly correlated among parents of cohort members in the Millennium Cohort Study. The two MCS questions, with headline associations with indications of anxiety and depression, are:

1. Compared to your friends, is your family richer, poorer or about the same? Richer, poorer, the same (reported by cohort member at age 11).
  - At age 14, prevalence of clinical levels of depression on the Short Moods and Feelings Questionnaire (SMFQ) [3] was 24.7% among those who reported that their family was poorer compared to 13.8% in those who reported their family to be richer.
  - At age 17, prevalence of clinical levels of distress on the Kessler 6 [57] scale was 25.3% among those who reported that their family was poorer compared to 13.8% among those who reported their family to be richer.
  - Prevalence of clinical levels of distress on the Kessler 6 scale among parents of cohort members was 12.8% for poorer families compared to 5.2% of richer families.
2. How well would you say you yourself are managing financially these days? (1) Living comfortably. (2) Doing alright. (3) Just about getting by. (4). Finding it quite difficult. 5 Finding it very difficult (reported by parent of cohort member at ages 9 to 14 years, with measures across years combined and grouped in quintiles).
  - At age 14, prevalence of SMFQ clinical levels of depression was 18.7% among the quintile managing least well compared to 11.7% in the quintile managing the best.
  - At age 17, prevalence of clinical levels of Kessler 6 distress was 18.9% among the quintile managing least well compared to 10.4% in the quintile managing the best.
  - Prevalence of Kessler 6 clinical levels of distress among parents of cohort members was 14.8% amongst the quintile managing least well compared to 1.0% in the quintile managing the best.

We also included a third question from Understanding Society which covers similar ground and will provide comparable subjective measures of SES.

3. On a scale of 1 to 7 where 1 = ‘completely dissatisfied’ and 7 = ‘completely satisfied], please tell me the number which you feel best describes how dissatisfied or satisfied you are with the income of your household. (1) Completely dissatisfied. {2) Mostly dissatisfied. (3) Somewhat dissatisfied. (4) Neither satisfied nor dissatisfied. (5) Somewhat satisfied. (6) Mostly satisfied. (7) Completely satisfied.

We have supplemented these measures with a range of questions based on job satisfaction and work environment, including autonomy and security. Proponents of UBI have suggested that these areas, in particular, should be impacted significantly by cash transfers that shift the balance of power away from employers and towards workers [51]. There are also indications from meta-analysis of a relationship between these areas and health, with strong correlations between job satisfaction and mental health, in particular [21]. We also included questions covering material deprivation and food security.

Finally, we included a question on care from the 2021 England and Wales Census. This is crucial, as the ability to undertake activity that is not traditionally remunerated is regarded both as a potential benefit of UBI [106], 24), an observed feature of previous trials [105] and an important issue in gender equality, as women are much more likely to undertake both paid and unpaid care ([106, 121], 23).

#### Self-reported health and wellbeing (direct health impacts)

Our assessment of associations between SES and mental wellbeing provided a foundation for development of the measures bank relating to self-reported measures of health and wellbeing. A range of mental wellbeing measures have been employed by large longitudinal cohort studies. For example: the Millennium Cohort Study includes the Short Moods and Feelings Questionnaire (SMFQ) [3] at 14, Kessler 6 [57] and the Warwick-Edinburgh Mental Well-being Scale (WEMWBS) [109] at 17, while parents answered the Malaise Inventory [95] when their child was 9 months old,Next Steps uses the General Health Questionnaire 12 (GHQ-12 [28] at 25,and Understanding Society employs GHQ-12 and Short-Form Health Survey (SF-12) [117], and WEMWBS (in particular waves). This does facilitate analysis of the measures most closely linked to clinical outcomes, but also means that many measures are only comparable through relatively complex, and sometimes insufficiently validated, calibration and mapping. Some of this work has been undertaken by McElroy et al. [70] with regard to mental wellbeing measures used in the six cohort studies managed by the Centre for Longitudinal Studies at UCL. It found that while some measures have good precision and reliability for assessing mental health at the high end of psychological distress other measures perform better at the lower end of and are more reliable at capturing wellbeing than distress. Further calibration work for measures at age 10/11 was undertaken by Gilbert et al. [26] and covers the longer SF-36 and WEMWBS. This study found that there was at least a ‘moderate-high correlation (> 0.60)’ between different measures, but this varied substantially ([26], 2) and leaves open questions about the degree to which data can be usefully compared and mapped so as to enable use in microsimulation modelling.

In terms of measures that enable clearer assessment of clinical mental health problems, the Patient Health Questionnaire (PHQ-9) [60] and Generalised Anxiety Disorder Assessment (GAD-7) [103] measure depression and generalised anxiety disorder according to DSM-IV symptoms. The short version PHQ-8 eliminates a question on self-harm. This is because it is not possible to guarantee support and safeguarding for respondents were they to report history of or plans to self-harm. For young people aged 8–16, we have proposed using RCADS [24] as a validated measure. While these measures have not been used in the major longitudinal studies under consideration, they are now the International Alliance of Mental Health Research Funders’ (IAMHRF) recommended measures for mental health and will likely be more widely used in future [22].

An alternative measure for adults is the Revised Clinical Interview Schedule (CIS-R) [63, 64]. There is a case to be made for the inclusion of CIS-R as it is the main measure used in the official mental health condition prevalence study in England [71] and has no licencing conditions. The measure used in the corresponding children and young people prevalence study (NHS [72] is the Development and Well-Being Assessment (DAWBA) [29, 125], which does have paid licence conditions (see ‘Discussion’ section below). While both can be completed through computerised versions, assessment of results by clinicians is still usually indicated. We have not recommended CIS-R and DAWBA over wholly self-reported alternatives as the latter would result in lower respondent load and administrative resource, are validated, and have sufficient sensitivity and specificity.

A number of self-reported measures of physical, or all-round, health are used in large cohort studies in the UK. Global self-rated health, broadly, ‘how is your health in general?’ usually with five options that vary between studies, is validated as an independent predictor of mortality and is very quick and easy to administer [38]. The version we selected is that used in the England and Wales Census, since it is the largest, most-comprehensive source of data available, but versions are included in most of the cohort studies, whether independently or as part of SF-12.

Measuring the impact of disability as defined by the social model, is also essential, and in keeping with Principle 6, as disabled people now comprised 21% of UK working-age people and 22% overall ([16], Table 4.1) in 2021 and face a range of intersectional determinants ([1, 92], 118–123). Disabled people are also disproportionately affected by welfare and reforms to welfare systems [47]. We have proposed the harmonised ONS version, as it is most-commonly used in national statistics (including the Census) and variations are included in major cohort studies.

We have supplemented these measures by including questions covering conditions diagnosed by a health professional and health service use from Understanding Society. There is strong evidence that the higher disease burden among people with lower SES is not matched by appropriately higher levels of diagnosis and treatment compared with higher SES individuals [107] and it is important to understand these access-to-healthcare issues and how they might be affected by cash transfers. In addition, it enables further analysis of how self-reported and physiological measures of health are associated with professional diagnosis.

Finally, with regard to subjective measures, we included the EQ-5D-5L [35] for adults and EQ-5D-Y for children and young people [120]. This enables a broader understanding of respondent health that can be monitored over time. While SF-12 [117] would provide a similarly broad assessment of health and has the benefits of being included in Understanding Society, it requires a paid licence that precludes its recommendation as a part of an Open Access resource (see ‘Discussion’ section below).

In terms of resourcing, we believe that we have been able to assemble a suite of questions that avoid the requirement of using in-person interviewers for the core measures and could be completed online, by post or by phone/video call. We propose, with regard to principles 5 and 6, that there should be both further testing and co-production of these self-reported measures during pre-study preparation as well as the provision of suitable alternatives, such as the option of a phone interview if required for access reasons, should they be required. Family Resources Survey data indicates that, in 2019/20, there were 1.6 million people in the UK with visual impairments, 3.5 million with dexterity impairments, 2.1 million with memory impairments and 1.8 million with learning disabilities ([16], Table 4.5). While there is overlap in these numbers, it is clear that if studies are to be truly representative of the public, accessible forms of participation must be available.

#### Physiological measures (direct health impacts)

Stress mitigation from cash transfers is a theoretical pathway in our model of impact (see [51]) but remains challenging to measure comprehensively. We include a subjective measure, the Perceived Stress Scale [11], in the self-report question bank. However, because individuals may perceive their level of chronic stress inaccurately [6] or self-report it differently for social reasons [101], we examined examples of biological material collection in studies such as Whitehall II and Understanding Society. The challenge of accurate measurement is not solely limited to stress. Chaparro et al. examined the associations between global self-rated health (SRH)—dichotomised to ‘good’ or ‘poor’—with biomarker indices, namely ‘visible weight-related’, ‘fitness’, ‘fatigue’, and ‘disease risk’ which reflected ‘different ways they may make the respondent feel and hence assess their health’ (2019, 2). They also assessed whether these associations are modified by age, gender, and/or socioeconomic position. They found that while self-rated global health is ‘overall strongly associated with objective measures of health’, ‘the strength of this association varies by the type of biomarker used as well as by gender, age, and income, though the latter to a lower extent than we hypothesised’ ([8], 9). They conclude that while ‘SRH is a valuable health indicator, caution should be taken when using SRH as the sole health measure when studying gender, age, and income health inequalities’ ([8], 9).

Given this background, Principle 5 and the additional ethical burden of biomarker collection in mind, we developed a module based on elements of major longitudinal cohort studies, particularly Understanding Society [42] along with others included in CLOSER [94] and Whitehall II [39, 61]. The majority of the physiological and recorded measures section should therefore be regarded as an optional add-on module, but one that deserves strong consideration, particularly for large studies. We have included evidence of association with health outcomes for each area measured and, as such, it is also possible to select from the bank based on particular interests within studies or where self-reported data is insufficient. The generic, adaptive protocol resource presents measures for trials and pilots for which linkage to Biobank or the new Our Future Health study would be possible.

#### Access and licensing conditions

Given Principle 5 and a general commitment to transformative science, we sought as fully as possible to produce Open Access protocols. While measures such as PHQ-9 and GAD-7 have no licence conditions attached to them, GHQ-12 [28] and SF-12 [117] require a paid licence in advance of use in studies, while EuroQol instruments (like EQ-5D) require licences that entail obligations for collaboration. WEMWBS also requires a licence, though conditions are relatively straightforward. Unpaid licences may be compatible with the spirit of Open Access collaboration, but paid licences pose ethical questions, particularly given the deployment of protocols for evaluation of trials intended specifically to mitigate health inequalities. Such interventions ought not to be compromised by the need to pay for survey measures, particularly where the validation process is unclear.

The case for payment lies in the quantity of existing data collected using paid measures that provides comparative, and microsimulation modelling, data for trial evaluations, as GHQ-12 and SF-12 have been deployed for over 10 years within Understanding Society. GHQ’s copyright holder also states that ‘part of the payment received from permissions is paid as a royalty to the Institute of Psychiatry to fund research’ [27]. While this may support scholarship, it is important to note that there have not been any major updates to the original English version of the measure since its introduction in the 1970s. For example, no child version has been developed by the copyright holder [27]. Similarly, SF-12, which is used as a measure of wellbeing by the UK Office for National Statistics, was released in 1996 with v2 in 2000 [65]. Again, the copyright holder has not developed a child version and there is no clear cost on their website [87]. As such, we sought to present Open Access alternatives. Our assessment identified a range of options that can be used in place of paid licence measures. In our measures bank, however, we highlight where paid alternatives with substantial comparative data can be sought where resources permit.

#### Trial duration and regularity of data collection

**Fig. 2:**
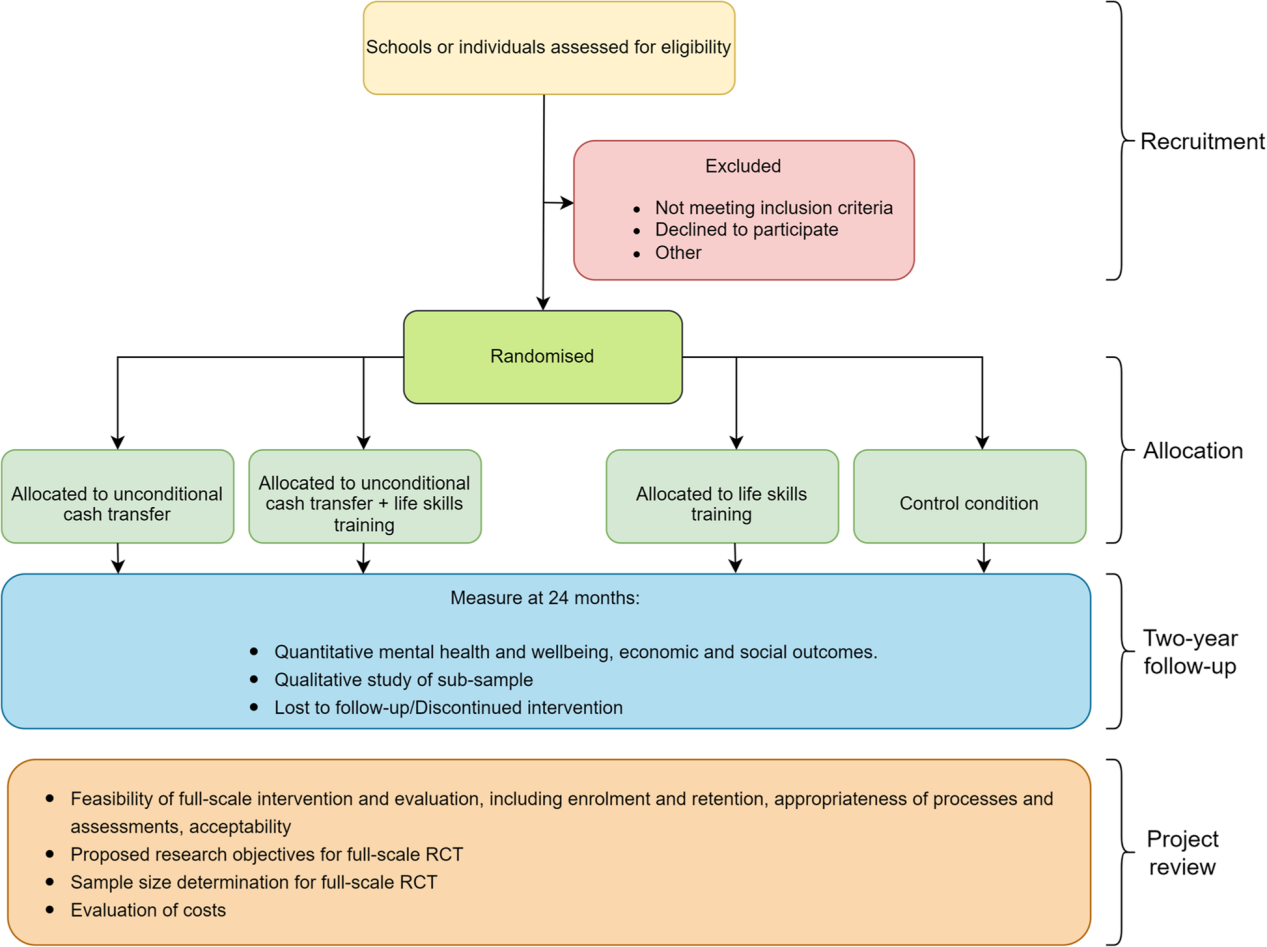
Adaptable study design for pilot intervention for young adults

It is important to provide examples of the types of trials and pilots to which this resource can be applied in order to demonstrate the ways in which the protocol can be adapted. A number of schemes have been designed for young people. The Welsh Government [118] has implemented its pilot of basic income for care leavers, while bases of similar schemes have been developed elsewhere [123]. Interest in this age group reflects concerns about the specific challenges posed in recent times to employment and independence, with cash transfers often proposed alongside life-skills support. These schemes are presented as means of supporting mental health, in particular [49], and mirror previous UK welfare interventions, such as the Educational Maintenance Allowance (EMA), which was intended to aid young people during a transitory period in their lives. In order to support such schemes, the resource features an adaptable study design for a pilot intervention for 18- to 20-year-olds (see Fig. 2).

Such schemes are necessarily time-limited, due to the ages of participants. Parameters for larger trials are less clear. Our model of impact indicates that pathways to health impact from cash transfers depend upon perception of material security and predictability that is unlikely to emerge during short trials and ‘micropilots’. However, we also note that a large intervention in the UK is likely only to be feasible within a period equal to a parliamentary electoral cycle, leaving, at most, three years for the intervention and evaluation ([53], 6). This is because, even if funding were provided privately, government departments, such as the Department for Work and Pensions, would be required to provide approval for payments with tax implications.

If negotiations can be undertaken with prospective governments ahead of elections to facilitate completion of design and contracting immediately following an election, it may then be possible to implement the cash-transfer intervention for the full 3 years, with principal data collection of health measures completed by the end of the second year. This would avoid measuring effects close to the ‘cliff-edge’ return to pre-intervention levels of income and conditionality among those in adulthood, though school leavers, for example, do not return to their pre-intervention condition because they would no longer be children. Such a duration would still not permit observation of longer-term social changes and the cascading impacts of, for example, participants returning to education. However, evidence from other projects, such as the negative income tax experiments of the 1970s ([25], e169) suggests that a 3-year study could provide indications of changes in health behaviours (e.g. [15]) as well as self-reported and physiological measures that can be used in microsimulation to estimate long-term health outcomes anticipated by the model of impact [25, 93].

## Discussion

Our work on the resource has highlighted the large number of measures employed to identify health impacts, the issues in their deployment to evaluate cash transfer trials and, perhaps most importantly, the need for standardisation of measures and new approaches to licencing. A key justification for the kinds of licenced measures that are currently relatively common in health studies is that ownership by organisations and paid licences facilitate the kind of resource-intensive validation, refinement and monitoring of impact that is necessary to ensure they remain relevant to the modern world. One body that licences materials, EuroQol, has invested effort in updating and maintaining its EQ-5D instruments, producing a revised version of the EQ-5D-3L [88], the EQ-5D-5L in 2011 [35], and a child version, the EQ-5D-Y, in 2010 [120]. This has been done while imposing no cost and one condition: that would-be users agree to collaborate with EuroQol researchers in large, > 100,000 participant, studies ([20], 6). Unfortunately, EQ-5D has been used neither in key studies of cash transfers nor major UK epidemiological datasets. Some copyright holders request payment for measures that have not been updated in decades or impose processes that render measures impractical to apply in all studies. For example, regarding GHQ-12, request for translation is subject to approval from the copyright holders, GL Assessment, which, if given, enables the would-be user to request translations separately from the MAPI Research Trust. The lack of public clarity on costs of licences is a significant obstacle to research. While CIS-R has no licencing conditions, use of the online DAWBA assessment tool appears to be charged at £10 per assessment [124] and it is unclear whether licencing conditions allow for administration of an independent online system.

To enable transformative research and data comparability between intervention evaluations and large cohort studies, it would be of substantial benefit for common measures, particularly those used for national statistics, to be brought into the public domain, either through purchase by institutions dedicated to Open Access or through creation and wholesale adoption of Open Access alternatives. Given the diversity of measures presently deployed in large datasets, there is also genuine need for data collected in calibration studies to be used to produce a tool that enables simple comparison between data collected via key measures, such as EQ-5D, SF-12, CIS-R, GHQ-12, and Kessler 6. This would be of substantial benefit both in prospective modelling of health outcomes from cash transfers and in assessment of the relationship between income and health more broadly.

We aim for this work to be a resource for two cash transfer studies currently under discussion, including the Welsh Government pilot of basic income for care leavers, with piloting and co-production essential to the design of the final protocols. Our hope, though, is that the resource will be used by other researchers and funders as the starting point for their own studies. It is only through this consistent and ongoing work that we will create data capable of assessing the health impact of cash transfer schemes and other socioeconomic interventions.

## Conclusion

The design of a generic, adaptive protocol resource for future use in cash transfer pilot and feasibility studies and trials is necessarily broad as studies will vary substantively depending on aims and resources. We have sought to put together a measures bank that will provide a much greater degree of comparability between data sources and a structure that facilitates use in microsimulation modelling. This provides an initial indication that health effects should and can be measured with valid and reliable brief instruments in surveys that must cover multiple topics.

The resource is intended as an initial step toward a fully validated system that assists in the design of pilot and feasibility studies and trials by researchers from a range of disciplines with an interest in health impact. It presents initial responses to a number of issues we have identified in the existing literature. These responses can only be examined further in co-production with representative participants and through implementation of the resource in pilot and feasibility studies and trials themselves. This process of development and implementation will support assessment of whether or not our proposed health outcome measures are acceptable, feasible and can be used with validity and reliability in the target population.

It is essential that specialists within the academic community work with members of the public to create protocols that produce widely accessible comparable data in pilot and feasibility studies and trials. Much greater collaboration, including through public funding of Open Access measures and integration of measures, is required to secure this outcome. We will continue to update the resource as our own work, and that of others, clarifies which measures are of most use, which do not work effectively at scale and where further improvements can be made. Updated versions will be made available at https://doi.org/10.17605/OSF.IO/FJH2P.

## Data Availability

Not applicable.
